# Ecological‐Linguistic Overlap: The Spatial Congruence Between Giant Panda (*Ailuropoda melanoleuca*) Habitats and Southwestern Mandarin

**DOI:** 10.1002/ece3.72821

**Published:** 2026-01-28

**Authors:** Shang Gao, Yiming Gao

**Affiliations:** ^1^ College of Ecology Lanzhou University Lanzhou China; ^2^ College of Wildlife and Protected Area Northeast Forestry University Harbin China

**Keywords:** *Ailuropoda melanoleuca*, biocultural conservation, ecological risk hypothesis, ecological‐linguistic overlap, Southwestern Mandarin

## Abstract

This study demonstrates significant ecological‐linguistic overlap between the distribution of the giant panda ( *Ailuropoda melanoleuca* ) and Southwestern Mandarin in China. Analysis of data from China's 4th National Giant Panda Survey reveals that 1745 of the 1864 recorded wild individuals (93.62%) reside in counties where Southwestern Mandarin is the predominant dialect. Spatial analysis confirmed a strong and statistically significant congruence (Schoener's *D* = 0.92, *p* = 0.04). Paleontological records further indicate that approximately 80%–90% of giant panda fossils are clustered within these linguistic zones. This ecological‐linguistic overlap may be attributed to shared environmental drivers and bamboo's dual ecological‐cultural role, providing a regional‐scale empirical case consistent with the core tenets of the ecological risk hypothesis. Our findings highlight the potential of linguistic maps as a supplementary layer of information for identifying conservation priorities.

## Graphical abstract

Our analysis reveals a significant spatial congruence between the distribution of giant pandas and Southwestern Mandarin in China. Based on the national census, 93.62% of wild pandas reside in counties where this dialect predominates, a pattern supported by strong statistical evidence (Schoener's *D* = 0.92). This ecological‐linguistic overlap underscores the role of shared environmental drivers and suggests linguistic maps could serve as a novel tool for informing conservation planning.

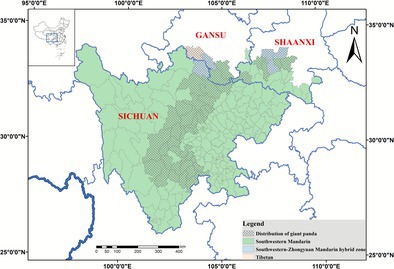

## 1 Introduction

The intricate relationship between biological and linguistic diversity has garnered increasing attention in recent years. Research has unveiled a striking spatial congruence between the two, hinting at an underlying functional link forged by environmental factors and historical contingencies (Hua et al. 2019; Harmon and Loh 2018; Turvey and Pettorelli 2014). This revelation holds profound implications for the development of conservation strategies, as it suggests that linguistic data could play a pivotal role in refining our understanding of species distribution and, consequently, inform more effective conservation efforts. Ecological factors such as temperature, humidity, topography, altitude, and vegetation are well‐established determinants of species and language richness at both community and system levels (Hawkins et al. 2003; Hof et al. 2012; Jetz et al. 2009). These factors act as limiting agents, dictating the distribution boundaries of specific species and languages. However, while macro‐level studies have established important general patterns of bio‐linguistic diversity correlations, they often mask region‐specific mechanisms and leave underexplored the relationships between particular species and specific linguistic groups.

The giant panda, a globally recognized flagship species for conservation, has been the subject of extensive research regarding its habitat. The role of shared ecological drivers is suggested by broad distributional overlaps among species within the same region. This is supported by previous research which found significant habitat overlap between the giant panda and sympatric species, including takin *(Budorcas taxicolor)*, Asiatic black bear *(Ursus thibetanus)*, and wild boar *(Sus scrofa)* within the panda's current range (Wang et al. 2015; Li and Pimm 2016). It is important to note that the panda's current distribution is a contracted state, resulting from both long‐term environmental constraints and profound anthropogenic pressures, such as historical deforestation and agricultural expansion (Luna‐Aranguré and Vázquez‐Domínguez 2021). Our study, however, focuses on identifying large‐scale biogeographic patterns that arise from the foundational environmental template shared by biological and cultural systems. If, as global patterns suggest, linguistic diversity is similarly shaped by these underlying environmental factors, then a congruence between giant panda distribution and the dominant linguistic group might be expected. We therefore designed this study to identify and test for a correlation with the specific language whose distribution aligns with the giant panda's range.

Southwestern Mandarin, the most geographically extensive and demographically dominant Chinese dialect, serves as a prime candidate for such analysis. Spatially concentrated across eight provinces and municipalities (Sichuan, Chongqing, Guizhou, Yunnan, Guangxi, Hubei, Hunan, and Shaanxi), with fragmented extensions in Gansu, Jiangxi and other provinces, this dialect group encompasses over 260 million speakers and is classified into six subregions (Chuanxi, Xishu, Chuanqian, Yunnan, Huguang, and Guiliu) based on phonological and lexical criteria (Xiong et al. 2012). Southwestern Mandarin spans biodiverse subtropical highlands that form the core habitat of giant pandas. However, despite the established global patterns of bio‐linguistic diversity correlations, the potential biogeographic correlation between the distribution of a flagship species like the giant panda and the spatial extent of a major linguistic group remains unexplored at regional scales. This study specifically investigates whether such a congruence exists between giant panda habitats and Southwestern Mandarin, providing a case study that bridges global patterns with regional specificity.

## 2 Materials and Methods

### 2.1 Giant Panda Distribution Data

Data on the current distribution of wild pandas were obtained from The Fourth National Survey Report on Giant Panda in China (National Forestry and Grassland Administration 2021), the fieldwork for which was conducted primarily between 2011 and 2014. The statistical unit for giant panda presence and population count is the administrative county. We acquired the vector boundaries for all counties in China from the Tianditu platform (https://www.tianditu.gov.cn), which utilizes the CGCS2000 geographic coordinate system. The panda distribution was thus georeferenced and visualized by shading the specific county polygons where giant pandas were recorded.

### 2.2 Southwestern Mandarin Distribution Data

The distribution of Southwestern Mandarin was primarily referenced from the Language Atlas of China (Xiong et al. 2012). The linguistic classification for each county was determined based on the predominant dialect type as defined in this and other authoritative dialectological studies. For counties located in transitional or hybrid zones, the prevailing dialect (e.g., Southwestern vs. Zhongyuan Mandarin) was ascertained through comprehensive phonological and lexical evidence cited in the literature. All spatial and statistical analyses are based on this final “prevailing language” classification. The complete set of source references justifying the classification for each county in our study area is provided in Table S1.

### 2.3 Paleontological Data

The distribution locations of extant giant panda fossils were sourced from Zhang et al. (Zhang et al. 2013). Spatial occurrence data were manually traced from images within the article, utilizing a unified geographic coordinate system to vectorize the image information and annotate relevant details.

### 2.4 Spatial Analysis and Statistical Testing

All spatial congruence and statistical analyses were conducted in R software. Schoener's *D* index was calculated based on binary classifications of panda presence (value of 1) or absence (value of 0) and Southwestern Mandarin distribution (1 for predominant, 0 otherwise) across the 49 counties constituting the panda's current range. The index ranges from 0 (no overlap) to 1 (complete overlap) (Schoener 1968). To evaluate the statistical significance of the observed D value, we performed a null model test with 10,000 permutations. The R code used for these analyses is available in the Supporting Information.

### 2.5 Visualization

**FIGURE 1:**
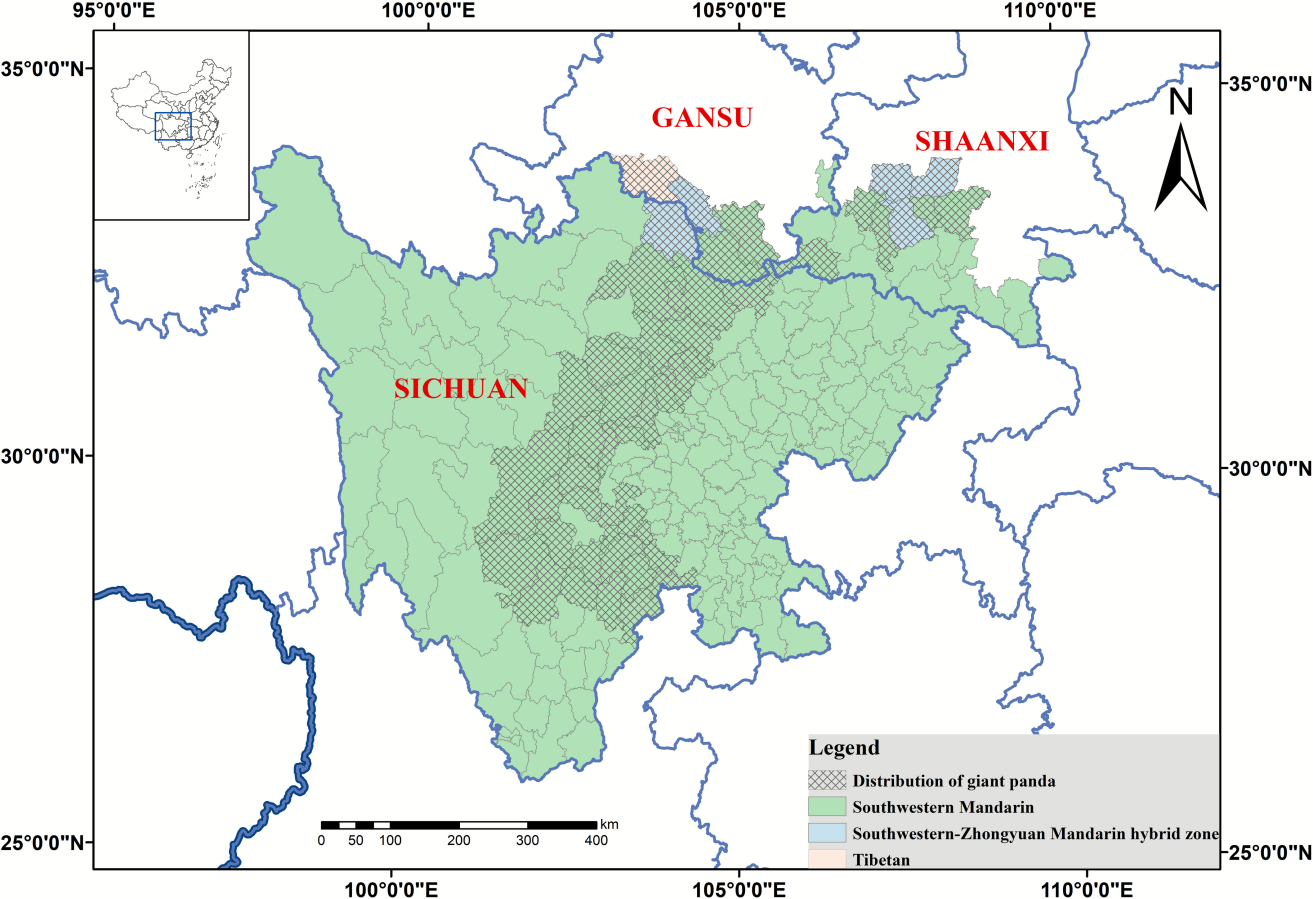
Spatial congruence between giant panda habitats and linguistic zones in Sichuan, Shaanxi, and Gansu provinces. The map shows the distribution of wild giant pandas (checkered pattern) overlaid on the full extent of Southwestern Mandarin and other language regions (Southwestern‐Zhongyuan Mandarin hybrid zone, Tibetan) within the three provinces that comprise the panda's entire range. This visualization reveals the pronounced spatial overlap between panda habitats and the Southwestern Mandarin linguistic area.

**FIGURE 2:**
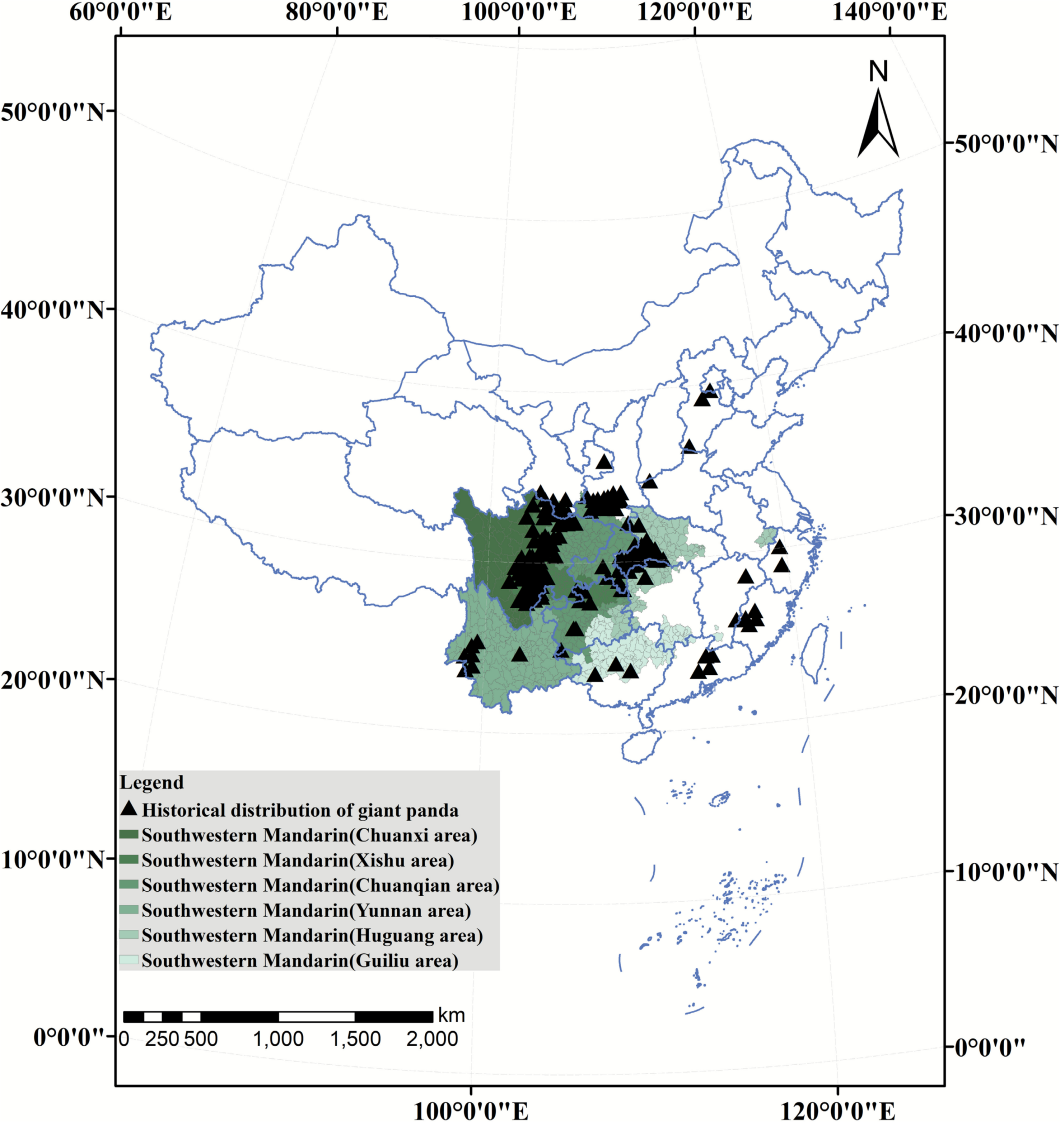
Distribution of Giant Panda Fossil Sites and Southwestern Mandarin in China. The map delineates the fossil records of the extant giant panda ( *Ailuropoda melanoleuca* ), represented by black triangles, alongside the distribution of Southwestern Mandarin. Six subdialect regions of Southwestern Mandarin are demarcated in varying shades of green. Fossil sites are primarily concentrated within the Southwestern Mandarin‐speaking regions, with sparse occurrences in other dialect areas.

All maps were created using ArcGIS 10.8 for visualization purposes. The geospatial data layers, including county boundaries, panda distribution polygons, and dialect region classifications, were projected into a unified coordinate system within ArcGIS to produce Figures 1 and 2.

## 3 Ecological‐Linguistic Overlap

The 4th National Survey Report on Giant Panda in China, provides a comprehensive overview of the extant wild giant panda population, which stands at 1864 individuals, distributed across Sichuan, Shaanxi, and Gansu provinces, encompassing 49 districts and counties (see Table S1 for detailed panda count and dialect data by county) (National Forestry and Grassland Administration 2021). Sichuan Province is home to the majority of the wild giant panda population, accounting for 74.4%, while Shaanxi and Gansu provinces contribute 18.5% and 7.1%, respectively. Given the established role of shared environmental drivers in shaping both biological and cultural distributions, we investigated the link between this biological pattern and linguistic zones. To investigate the potential link between this biological distribution and linguistic patterns, our analysis revealed a strong spatial congruence between the distribution of giant pandas and Southwestern Mandarin. Of the 1864 wild giant pandas recorded in the census, 1745 individuals (93.62%) reside within the 45 counties classified as Southwestern Mandarin predominant. To quantitatively evaluate this spatial congruence, we employed Schoener's *D* index based on binary classifications of panda presence/absence and Southwestern Mandarin distribution (including hybrid zones where Southwestern Mandarin was the prevailing language). Results confirmed a strong and statistically significant spatial overlap between giant panda distribution and Southwestern Mandarin (*D* = 0.92). Null model testing (10,000 permutations) confirmed that this observed congruence significantly exceeds random expectations (null mean = 0.88 ± 0.02 SD, *p* = 0.04, *Z* = 2.55). The results demonstrate a strongly nested spatial pattern, with panda populations almost entirely confined to counties where Southwestern Mandarin is predominant.

Linguistically, Sichuan Province is predominantly characterized by the use of Southwestern Mandarin, with the exception of certain ethnic minority languages such as Tibetan, Yi, and Qiang spoken in the western part of the province. In contrast, Zhongyuan Mandarin and Lan‐Yin Mandarin are the dominant languages in most areas of Shaanxi and Gansu Provinces (Xiong et al. 2012; Chao and Wen 2013). However, a notable pattern emerges in the districts and counties where pandas are found within these two provinces: the prevailing language is Southwestern Mandarin or a hybrid thereof. Dialectological studies further corroborate this pattern, highlighting the presence of varying degrees of Southwestern Mandarin characteristics in the local dialects of Taibai, Yangxian, and Zhouzhi in Shaanxi Province, and Zhouqu in Gansu Province, in terms of phonology, lexicology, and syntax.

Beyond the current distribution across Sichuan, Shaanxi, and Gansu, paleontological records of the giant panda ( *Ailuropoda melanoleuca* ) reveal a broader historical range. An analysis of fossil records indicates that approximately 80%–90% of known fossil sites for the extant species are located within provinces that are now classified as Southwestern Mandarin‐speaking regions (including Hubei, Guizhou, Yunnan, Chongqing, and Guangxi), while sparse occurrences have been documented in northern and southeastern provinces (see Table S2 for detailed fossil counts by province) (Zhang et al. 2013). This historical biogeographic pattern aligns with the contemporary linguistic‐ecological overlap observed in wild populations. This congruence implies that key environmental drivers, including topographic complexity and humid subtropical climates, have jointly constrained both giant panda habitat suitability and human linguistic diversity over millennia.

## 4 Discussion

### 4.1 Ecological Risk Hypothesis

The striking spatial congruence between giant panda distribution and Southwestern Mandarin (93.62% overlap, Schoener's *D* = 0.92) invites interpretation through the lens of the ecological risk hypothesis. This hypothesis posits that environmental uncertainty and resource acquisition risks fundamentally shape human sociocultural adaptations, including linguistic patterns, as mechanisms to buffer against ecological challenges (Coupé et al. 2013). Our findings provide compelling empirical support for this view by demonstrating how shared environmental drivers constrain both biological and cultural distributions. Specifically, factors such as temperature, humidity, topography, elevation, and vegetation, which are well‐established constraints on species distribution (Ember and Ember 2007; Everett et al. 2015), also shape the northern boundaries of the Southwestern Mandarin region. The complex topography and specific climatic regimes of the subtropical highlands, which delineate suitable giant panda habitat, simultaneously presented historical human populations with distinct ecological risks and opportunities. This shared environmental template fostered adaptations in both the flagship species and the dominant human linguistic group. While recent anthropogenic pressures have undoubtedly contracted the panda's range, our paleontological evidence indicates that the core congruence with Southwestern Mandarin is a long‐standing pattern. This finding suggests that the shared environmental template established a persistent biogeographic correlation, which has subsequently been modulated by more recent anthropogenic processes.

Critically, the distribution and abundance of key resources within this shared environment served as a mediator of these risks. In the case of the giant panda and Southwestern Mandarin, bamboo serves as a pivotal link. The survival of pandas is inextricably linked to bamboo, which constitutes the bulk of their diet and provides essential habitat elements. Similarly, the people of southwest China have developed a profound practical and cultural reliance on bamboo. Historically, bamboo has been applied as a multifunctional resource across diverse areas, from fundamental uses in clothing, food, shelter, and transportation to the manufacture of items like arrowheads and writing scrolls, and to critical applications in construction (Dlamini et al. 2022). However, this reliance can also lead to conflict when resource use becomes unsustainable. For instance, intensive bamboo shoot harvesting by Yi communities in areas like the Mabian Dafengding Reserve represents a significant conservation challenge, where overharvesting creates direct competition with panda populations (Li 2025). This instance of conflict reveals that the broad ecological‐linguistic overlap also contains microlevel competitions for resources.

On a deeper cultural level, the people of southwest China exhibit a strong bamboo worship consciousness, which is evident in the rich tapestry of poetic works that celebrate this versatile plant. This cultural profundity is epitomized by the enduring Chinese idiom “The Complete Bamboo in The Breast” (胸有成竹), a concept famously articulated by the Northern Song literatus Su Shi, which describes a state of perfect mental preparation and internalization (Fuller 1993). The same poet further exalted the spiritual value of bamboo in daily life, declaring, “Better to eat without meat than live without bamboo” (宁可食无肉，不可居无竹). This explicit privileging of bamboo's spiritual sustenance over material nourishment highlights its deep‐seated role in shaping human sensibility and cultural identity within this ecoregion.

Thus, bamboo represents not only a shared keystone resource but also the focal point around which ecological pressures and human cultural (including linguistic) adaptations have converged. This convergence of ecological and cultural aspects around bamboo underscores the complex interplay between the natural environment and human societies. The case of the giant panda and Southwestern Mandarin exemplifies a broader pattern of coadaptation to montane environments, which foster both high biological and cultural diversity, consistent with global observations (Stepp et al. 2005). This, in turn, emphasizes the need for integrated conservation strategies that consider both biological and cultural dimensions.

### 4.2 Conservation Implications

The observed distributional congruence between the giant panda and Southwestern Mandarin offers a novel perspective for biocultural conservation. While the primary tools for conservation should rely on high‐resolution ecological data, habitat modeling and field surveys, our findings suggest that linguistic maps could serve as a supplementary source of information in regional‐scale assessments. This potential utility is grounded in the shared environmental drivers, most notably complex topography and humid subtropical climates. These factors have historically shaped both the panda's habitat and long‐term human settlement patterns, making Southwestern Mandarin a cultural marker of these specific ecoregional conditions. Thus, in contexts of broad‐scale conservation prioritization or in data‐limited regions, linguistic maps might be considered as one exploratory indicator among others, helping to contextualize or generate hypotheses about areas where long‐term human‐environment interactions align with key panda habitat conditions. In this way, the distribution of languages, shaped by long‐term environmental adaptation, can provide a distinct and complementary line of evidence for understanding the ecological constraints that also shape species distributions. This approach underscores the value of integrating biocultural perspectives into conservation science, not as a replacement for ecological data, but as a complementary lens for understanding coupled human‐natural systems.

In light of these findings, future research should test the predictions of the ecological risk hypothesis through detailed quantitative analyses. A valuable direction would be to empirically examine the association between key environmental variables (e.g., topography, climate) and the distributions of both giant panda presence and linguistic features. Such research would help explore whether linguistic data can consistently inform conservation efforts in other contexts, potentially contributing to the development of more comprehensive and effective conservation strategies. In conclusion, the distributional consistency between the giant panda and Southwestern Mandarin suggests a novel perspective for conservation planning, illustrating how linguistic data might support the formulation of targeted strategies for this iconic species and other species within comparable biocultural landscapes.

## Author Contributions

**Shang Gao:** conceptualization (lead), formal analysis (equal), investigation (equal), methodology (lead), supervision (lead), writing – original draft (lead), writing – review and editing (lead). **Yiming Gao:** conceptualization (supporting), data curation (lead), formal analysis (equal), investigation (equal), methodology (supporting), visualization (lead), writing – original draft (supporting), writing – review and editing (supporting).

## Conflicts of Interest

The authors declare no conflicts of interest.

## Supporting information


**Table S1:** County‐level dataset of giant panda presence and prevailing language classification.
**Table S2:** Historical records on distributions of giant pandas.

**Data S1:** ece372821‐sup‐0002‐Supinfo.R.

## Data Availability

All the required data are uploaded as Supporting Information.
